# Annexin A1 Deficiency does not Affect Myofiber Repair but Delays Regeneration of Injured Muscles

**DOI:** 10.1038/srep18246

**Published:** 2015-12-15

**Authors:** Evgenia Leikina, Aurelia Defour, Kamran Melikov, Jack H. Van der Meulen, Kanneboyina Nagaraju, Shivaprasad Bhuvanendran, Claudia Gebert, Karl Pfeifer, Leonid V. Chernomordik, Jyoti K. Jaiswal

**Affiliations:** 1 Section on Membrane Biology, Program of Physical Biology, Eunice Kennedy Shriver National Institute of Child Health and Human Development, National Institutes of Health, Bldg. 10/Rm. 10D05, 10 Center Dr. Bethesda, Maryland 20892-1855, USA; 2 Children’s National Medical Center, Center for Genetic Medicine Research, 111 Michigan Avenue, NW, Washington DC 20010-2970, USA; 3 Section on Genome Imprinting, Program on Genomics of Differentiation, Eunice Kennedy Shriver National Institute of Child Health and Human Development, National Institutes of Health, USA; 4 Department of Integrative Systems Biology, George Washington University School of Medicine and Health Sciences, Washington DC, USA

## Abstract

Repair and regeneration of the injured skeletal myofiber involves fusion of intracellular vesicles with sarcolemma and fusion of the muscle progenitor cells respectively. *In vitro* experiments have identified involvement of Annexin A1 (Anx A1) in both these fusion processes. To determine if Anx A1 contributes to these processes during muscle repair *in vivo*, we have assessed muscle growth and repair in Anx A1-deficient mouse (AnxA1−/−). We found that the lack of Anx A1 does not affect the muscle size and repair of myofibers following focal sarcolemmal injury and lengthening contraction injury. However, the lack of Anx A1 delayed muscle regeneration after notexin-induced injury. This delay in muscle regeneration was not caused by a slowdown in proliferation and differentiation of satellite cells. Instead, lack of Anx A1 lowered the proportion of differentiating myoblasts that managed to fuse with the injured myofibers by days 5 and 7 after notexin injury as compared to the wild type (w.t.) mice. Despite this early slowdown in fusion of Anx A1−/− myoblasts, regeneration caught up at later times post injury. These results establish *in vivo* role of Anx A1 in cell fusion required for myofiber regeneration and not in intracellular vesicle fusion needed for repair of myofiber sarcolemma.

Healing injured muscle involves relatively fast (minutes) repair of the injured myofiber cell membrane by fusion of intracellular membrane with the cell membrane^1^. A fiber that fails to repair degenerates and is subsequently regenerated over multiple days by a process that involves myogenic differentiation of satellite cells, the muscle-specific stem cells, into myoblasts. The myoblasts fuse with each other and with existing myofibers regenerating the damaged myofiber. Thus, the processes of myofiber repair and regeneration both require proteins that facilitate membrane fusion. While a number of proteins involved in these membrane fusion processes have been identified^2^,^3^,^4^,^5^,^6^,^7^,^8^,^9^,^10^, the molecular mechanisms underlying fusion processes leading to repair or regeneration of injured mammalian myofiber remain to be understood.

Annexin protein family is known to facilitate membrane fusion and has gained wide recognition for their involvement in sarcolemmal repair^11^,^12^. Several recent studies have also documented dependence of myogenic differentiation and *in vitro* myoblast fusion on annexin proteins, including annexin A1, Anx A1^6^,^13^,^14^. Anx A1 has been shown to facilitate cell membrane repair *in vitro*, where it accumulates at the injured cell membrane^12^,^15^,^18^. Cytosolic and extracellular Anx A1, an important regulator of innate and adaptive immunity^19^, regulates proliferation, differentiation and migration of different cells^10^; and interacts with the sarcolemmal repair protein dysferlin^12^,^16^,^20^,^21^. Following *in vivo* muscle injury, Anx A1 expression increases concomitant with the appearance of the first new multinucleated myotubes^22^(Public Expression Profiling Resource at http://pepr.cnmcresearch.org/). Despite the *in vitro* evidence of a role of Anx A1 in membrane fusion process needed for myofiber repair and regeneration, Anx A1-knockout mice (AnxA1−/−) display no gross abnormalities^23^. However, the effect of Anx A1 deficit on muscle function, repair and regeneration *in vivo* has not been investigated.

In this study, we focused on the effects of Anx A1 deficiency on the growth and physiology of mammalian skeletal muscles. We observed no detectable differences in muscle between Anx A1−/− and the parental (C57Bl6) wild type (w.t.) mice. Both sets of adult animals have similar body weight and weight of specific muscles. We observed a small reduction in contractile force of the fast twitch (*Extensor Digitorum Longus,* EDL) muscle, but not of the slow twitch (soleus) muscle. Despite the reduced contractile force of the fast twitch muscle, we did not observe any deficit in the ability of the fast twitch muscles from the Anx A1−/− mice to undergo repair following focal sarcolemmal injury or following lengthening contraction injury. Similar to our observation *in vitro* that fusion between Anx A1−/− myoblasts is slower at earlier times but then catches up^6^, we find that the lack of Anx A1 resulted in a significant delay in the muscle regeneration after notexin-induced injury *in vivo*. This delay in muscle regeneration in Anx A1−/− animals was not associated with deficits in post-injury activation and myogenic differentiation of satellite cells, but with a delay in fusion of activated myoblasts with the regenerating myofibers. Our results substantiate the hypothesis that Anx A1 is involved in cell-cell fusion during myofiber regeneration following acute muscle injury *in vivo* but not in muscle development or repair of injured myofiber sarcolemma.

## Results

### Anx A1 deficit does not alter muscle size and histology

**Figure 1:**
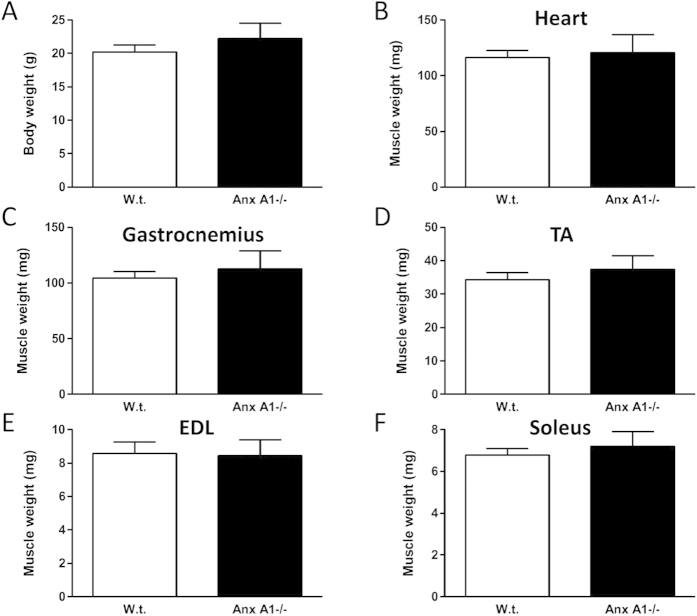
Annexin A1 deficiency does not affect body and muscle weight. (**A**) Body weight of 4–8 months old adult animals (n = 7 for w.t. and n = 4 for Anx A1−/−). (**B–F**) Freshly isolated muscles for the heart and each of the limb muscles were weighed (n ≥ 8 skeletal muscles for each genotype). All data are expressed as means ± S.E.M. and all the differences between the w.t. and Anx A1−/− muscles were statistically insignificant (p > 0.4).

**Figure 2:**
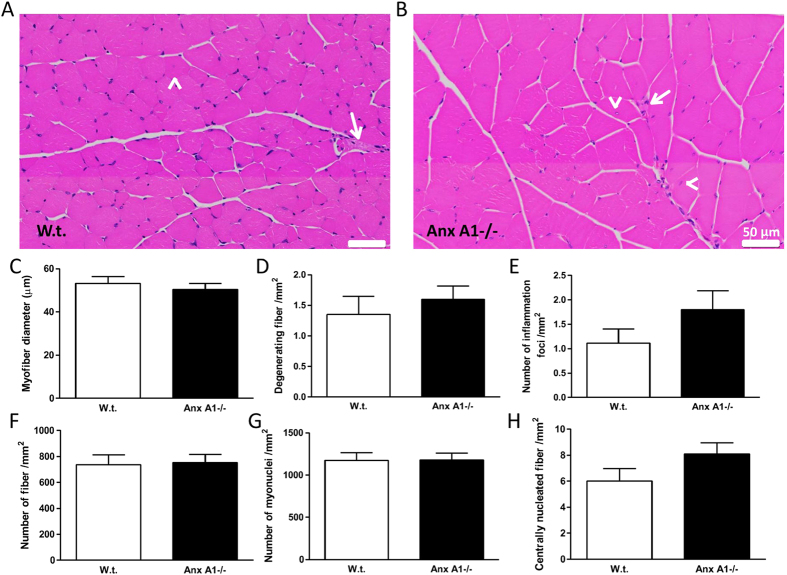
Lack of annexin A1 does not lead to histopathology of *Gastrocnemius* muscle. Paraffin embedded muscle from 4 independent animals were sectioned and stained with Hematoxylin and Eosin. The entire stained sections were imaged using digital slide scanner using 40x objective. (**A,B**) Representative histological images of the muscle from a w.t. and an Anx A1−/− mice. Scale bar 50 μm. White arrow shows degenerating fiber and white arrowheads show central nuclei marking the recently regenerated fiber. (**C**) Minimal Feret’s diameter of myofibers in the muscles from w.t. and Anx A1−/− mice. (**D**) Number of degenerating fiber in the muscle in w.t. and Anx A1−/− mice. (**E)** Number of inflammatory foci in the muscle in w.t. and Anx A1−/− mice. (**F**) Number of fiber in the muscle, **G.** Total number of myonuclei, and (**H**) Number of centrally nucleated fibers in the muscle from w.t. and Anx A1−/− mice. All data shown in C-H are expressed as means ± S.E.M. n = 4 mice. All the differences between w.t. and Anx A1−/− data are statistically insignificant (p > 0.05).

Since Anx A1 was shown to be involved in myoblast fusion *in vitro*^6^,^13^,^14^, we assessed the effect of Anx A1 deficit on the muscle using Anx A1−/− and w.t. mice. Similar to what was previously reported^23^, we found that body weight of Anx A1−/− mice was indistinguishable from that of w.t. mice, and observed no difference in the weight of the specific muscles including heart, *Soleus*, EDL, *Tibialis Anterior* (TA) and *Gastrocnemius* (Fig. 1). Histological examination of H&E stained *Gastrocnemius* muscles from w.t. and Anx A1−/− mice (Fig. 2A,B) identified no significant difference in the myofiber diameter (Fig. 2C), extent of skeletal myofiber degeneration (Fig. 2D), and the number of inflammatory foci (Fig. 2E). The total number of myofibers or myonuclei per unit area (Fig. 2F,G), and the number of regenerating fibers (identified as centrally nucleated fibers – Fig. 2H) showed no difference between w.t. and Anx A1−/− mice. These results indicated that not only does lack of AnxA1 has no effect on body growth and growth of the skeletal muscles, it also does not cause muscle degeneration or increase regeneration.

**Figure 3:**
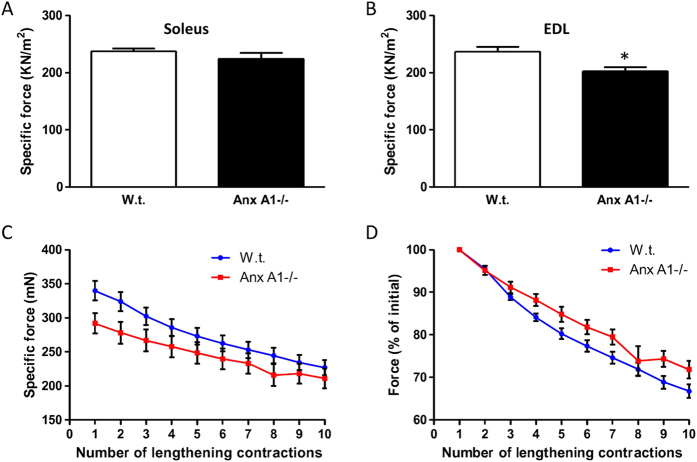
Annexin A1 deficit reduces EDL contractile force but not the ability to recover from lengthening contraction injury. Specific force generated by freshly isolated (**A**) Soleus and (**B**) EDL muscles was measured for w.t. (n = 8 muscles) and Anx A1−/− (n = 6 muscles). (**C**) Loss in muscle force as a result of repeated 10% lengthening contractions of muscles from w.t. (n = 8 muscles) and Anx A1−/− (n = 6 muscles) mice. (**D**) The data presented in C are normalized to the initial forces. All data are expressed as means ± S.E.M. The samples were compared by two-tailed unpaired t-test analysis and level of statistical significance of differences is shown as * for p ≤ 0.05 and rest of the samples were found to be not significant (p > 0.05).

Next, to examine if lack of Anx A1 affects muscle function *in vivo*, we examined the ability of Anx A1−/− muscle to generate contractile force. For this we isolated a slow twitch (soleus) and a fast twitch (EDL) muscle from w.t. and Anx A1−/− mice and measured the ability of these muscles to generate contractile force following electrophysiological stimulus. Lack of Anx A1 did not affect the contractile force generated by the soleus muscle (Fig. 3A), while the EDL muscle showed a reduced specific force as compared to the w.t. mice (Fig. 3B).

### Anx A1-deficient myofibers repair efficiently from focal and mechanical injury

**Figure 4:**
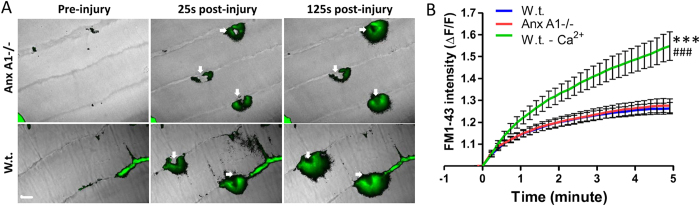
Repair of sarcolemmal injury is not affected by Annexin A1 deficiency. (**A**) Time-lapse images of myofibers of freshly isolated w.t. or Anx A1−/− biceps muscle before as well as 25 and 125 seconds after laser injury (white arrow indicates site of injury). Entry of dye into the myofiber is shown by the overlay of FM dye fluorescence (green) on the bright field (grayscale) image of the myofibers. (**B**) Quantification of FM1-43 influx, following laser injury into fibers isolated from w.t. in presence or absence of calcium (n = 19 fibers in presence and n = 10 in absence) and Anx A1−/− in presence of calcium (n = 20 fibers) from 2 mice for each condition. The plots show FM dye intensity averaged for all the fibers at indicated times following injury. The value at each time point is normalized to the dye intensity for the fiber at the time of injury (F0) and the error bars represent SEM. Scale bar 10 μm. Statistical difference between the kinetics of increase in averaged FM dye intensity were compared by two-tailed unpaired t-test and statistical significance of the difference in kinectis is shown as *** for p ≤ 0.001 compared to w.t. and ^###^ for p < 0.001 compared to Anx A1−/−.

In view of the lowered contractile force of the EDL muscle and the previously reported involvement of Anx A1 in repair of injured plasma membrane of cultured cells^15^,^24^,^25^, we hypothesized that the EDL myofibers have reduced ability to repair following acute injury. To test this, in the first approach, we injured the myofibers using stretch-induced injury. The loss in contractile force following each round of lengthening contraction-induced injury of Anx A1−/− EDL muscle was similar to that of the w.t. EDL muscle indicating that lengthening contraction does not damage the AnxA1−/− muscle more than the w.t. muscle (Fig. 3C). This similarity in lengthening contraction injury-induced decline in EDL muscle contractile force was even more obvious when the contraction-induced change in specific force was normalized to the contractile force prior to stretch induced injury (Fig. 3D). As lengthening contractions cause diffuse injury to sarcolemma as well as to myofibrils, in the next approach we directly monitored the kinetics of sarcolemmal repair in individual myofiber by controlled focal injury of the sarcolemma using a 10ms pulse of a focused laser beam as previously described^17^. Again, we injured myofibers in a fast twitch muscle (Biceps) and monitored repair by quantifying the influx of the membrane impermeant fluorescent probe (FM1-43) into the myofiber^17^. We have previously demonstrated the utility of this approach to monitor poor repair ability of biceps myofiber from dysferlin-deficient mice^17^. Here we observed that the sarcolemma of Anx A1−/− biceps myofibers repaired from focal injury with the kinetics that was indistinguishable from that of the w.t. myofibers injured in the presence of calcium (Fig. 4A,B). Further, the efficient repair kinetics for both Anx A1 −/− and w.t. myofibers injured in the presence of calcium was evident when compared to the w.t. myofibers that failed to repair due to injury in the absence of extracellular calcium (Fig. 4B). Thus, using two independent acute myofiber injury approaches in intact muscles (not isolated myofibers) we found that there was no detectable difference in the repair ability of the Anx A1-deficient myofibers. These results were in agreement with the lack of any histopathology in the Anx A1-deficient skeletal muscle (Fig. 2).

### Anx A1 deficiency slows down muscle regeneration

Since Anx A1 plays an important role in myoblast fusion *in vitro*^6^ and is up-regulated during myogenic fusion stage of muscle regeneration^22^, we next examined the effects of Anx A1 deficiency on myoblast fusion *in vivo*. To induce regeneration, we acutely and focally damaged the TA muscle by a notexin injection. Notexin selectively damages the myofibers, triggering a focal muscle necrosis and regeneration. Macrophages infiltrate the injured site to remove necrotic debris, which is followed by the activation of myogenic cells required for muscle regeneration^26^,^27^. Post injury regeneration involves proliferation of the satellite cells underneath the basal lamina. Thus after notexin injection, the muscle was allowed to regenerate while the mice were provided bromodeoxyuridine (BrdU) to label the newly synthesized DNA of dividing cells, resulting in their nuclei being labeled with BrdU. Proliferation and differentiation of satellite cells is followed by cell fusion to generate new myofibers with centrally located nuclei that upon myofiber maturation move to the periphery.

**Figure 5:**
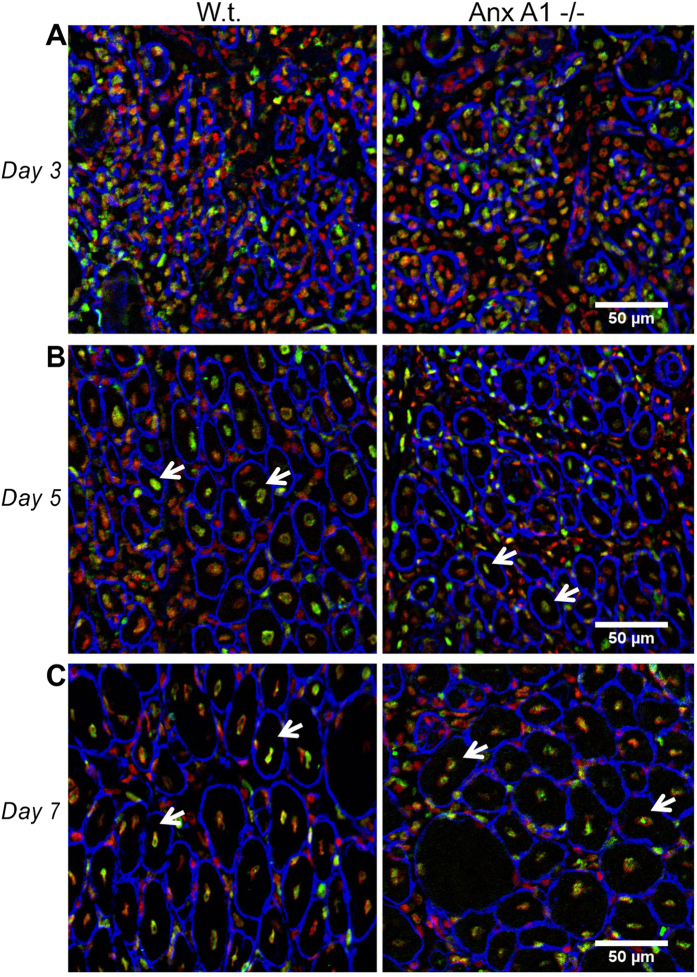
Sections of the injured and regenerating TA muscle taken from w.t. and Anx A1−/− mice at different time after notexin injection. Sections taken 3, 5 and 7 days post-injury (**A**–**C**, respectively) were labeled with antibodies to laminin (blue) and to BrdU (green) and with propidium iodide (red) to mark myofiber borders, nuclei of newly divided cells, and all cell nuclei, respectively. Shown are representative fields of view in the vicinity of the injection site. Arrows mark some of the regenerating myofibers identified as myofibers with centrally located BrdU-labeled nuclei. Scale bar 50 μm.

In the muscle sections at days 3, 5 and 7 after the notexin injury we observed both unregenerated necrotic regions and regenerating myofibers (Fig. 5). In the muscle sections at 3 days after notexin-induced injury we observed large unregenerated regions infiltrated with macrophages that were positive for the pan-macrophage marker F4/80 (Supplementary Fig. 1). Anx A1 deficiency did not affect the density of macrophages in the unregenerated injured region - 3100 ± 400 macrophages/mm^2^ vs. 3200 ± 200 macrophages/mm^2^ (n = 3 mice each). Poorly defined borders of the myofibers (identified by the basement membrane staining with anti-laminin antibody (Fig. 5A)) hindered quantitative analysis of the regenerating myofibers in these day 3 muscle sections. Thus we focused our subsequent analysis of muscle regeneration on the sections taken from days 5 and 7 after notexin injury.

**Figure 6:**
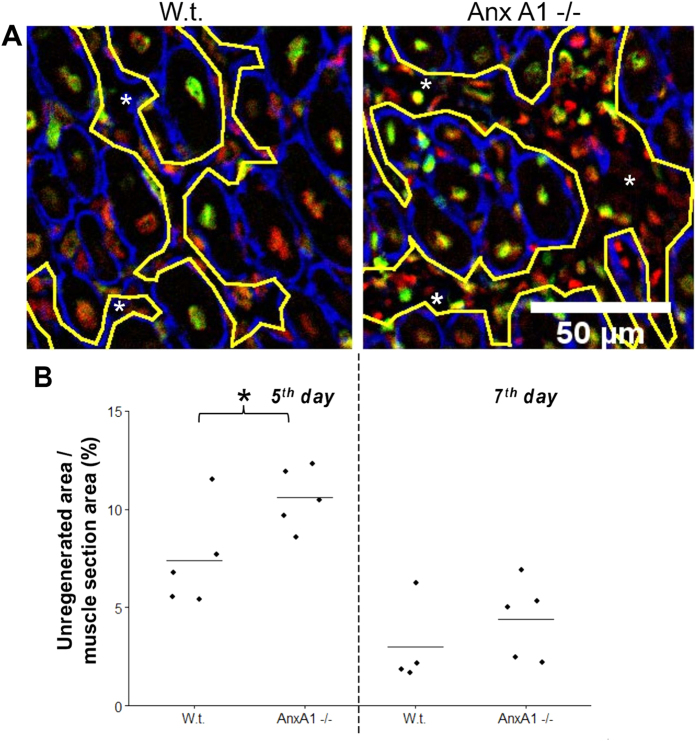
Anx A1 deficiency increases area of unregenerated regions in the vicinity of the injury site observed 5 days post injury. (**A**) Images of the sections taken from w.t. and Anx A1−/− mice 5 days after notexin injection and labeled as in Fig. 5. Yellow lines show the borders of unregenerated (necrotic) regions (marked by *). Scale bar 50 μm. (**B**) Unregenerated (necrotic) regions were identified in muscle sections taken from w.t. and Anx A1−/− mice 5 or 7 days after notexin injection (n = 4 for w.t. mice at 7 day post-injury and n = 5 for all other conditions). We normalized the total unregenerated area in each analyzed field of view to the area of the muscle section in this field and present the means for each mouse in a given condition with a line within the group of the points to show the mean of the means for all mice in a given condition. Level of statistical significance of differences between w.t. and Anx A1-deficient mice is shown as * for p ≤ 0.05 and the rest of the samples were not significantly different from each other, p > 0.05.

**Figure 7:**
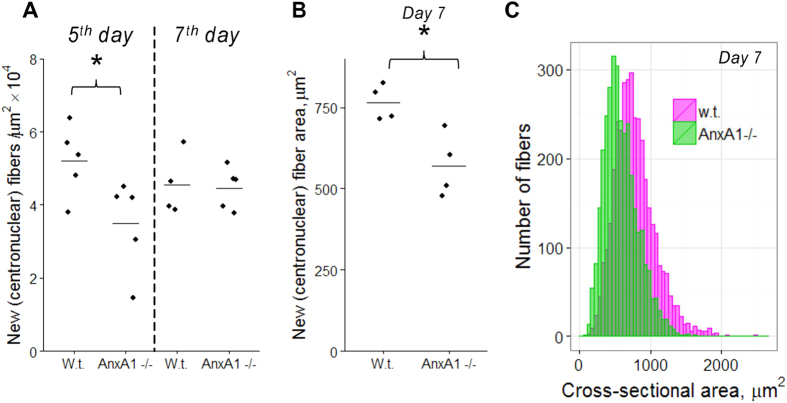
Anx A1 deficiency lowers mean number and cross section area of myofibers with centrally located BrdU-labeled nuclei in the vicinity of the injury site. (**A**) New fibers were identified and counted in muscle sections taken from w.t. and Anx A1−/− mice 5 or 7 days after notexin injection (n = 4 w.t. mice at 7 days post injury and n = 5 mice for each of the other conditions). B, C New fibers were identified and their cross-section areas measured for 4 Anx A1−/− mice and 4 w.t. mice at 7 days post injury. (**C**) All fiber cross-section areas for Anx A1−/− mice were pooled together (n = 3266, the distribution shown in pink) and compared with the data pooled together for all w.t. mice (n = 3646, the distribution shown in green). (**A**,**B**) The points are the mean numbers of new fibers in 1 μm^2^ of section area (**A**) and the mean cross section areas of new fibers for each mouse in given condition and a line within the group of the points shows the mean of the means for all mice in a given condition. (**A**,**B**) Level of statistical significance of differences between w.t. and Anx A1-deficient mice is shown as * for p < 0.05, the rest of the samples were not significantly different from each other, p > 0.05.

In the sections of muscles 5-day post notexin injection, we observed the laminin-enclosed regenerating myofibers that were always larger than 100 μm^2^ and had centrally located nuclei labelled with antibodies to BrdU (“new myofibers”), while there were still significant unregenerated, disorganized regions between the fibers (Figs 5,6). These unregenerated regions in the vicinity of the injury site were greater in AnxA1−/− muscle sections as compared to the muscle sections from the w.t. mice (Fig. 6). These Anx A1−/− muscle sections also had fewer new myofibers as compared to the w.t. (Fig. 7A). Note that our analysis would possibly miss regenerating fibers that had not yet formed a basement membrane around them. The differences between the sections taken from w.t. and Anx A1−/− mice in the areas of unregenerated regions and in the number of new myofibers disappeared by 7 days after injury (Figs 6 and 7A). However, even at this time, the average area of the new fibers in Anx A1−/− mice remained smaller than that in w.t. mice (Fig. 7B,C).

Myoblasts that fuse into newly regenerated myofibers are generated by proliferation and subsequent myogenic differentiation of satellite cells. To assess if Anx A1 deficit affects number of activated satellite cells, we identified cells expressing very early myogenic marker Myf5^28^ in the sections of the regenerating muscles at 5 days after injury (Supplementary Fig. 2). Muscle sections from two Anx A1 −/− mice showed 117 ± 7 Myf5-positive cells/mm^2^ vs. 120 ± 10 cells/mm^2^ in the muscle sections from 3 w.t. mice. To compare, the number of Myf5 positive cells in uninjured regions of the muscle section from 3 w.t. mice was 10 times lower (10 ± 0.7 cells/mm^2^). Similar numbers of Myf5 positive cells in Anx A1−/− and w.t. muscles show that a deficit in injury-triggered satellite cell activation is not the basis for delayed regeneration of notexin-injured AxnA1−/− muscles.

**Figure 8:**
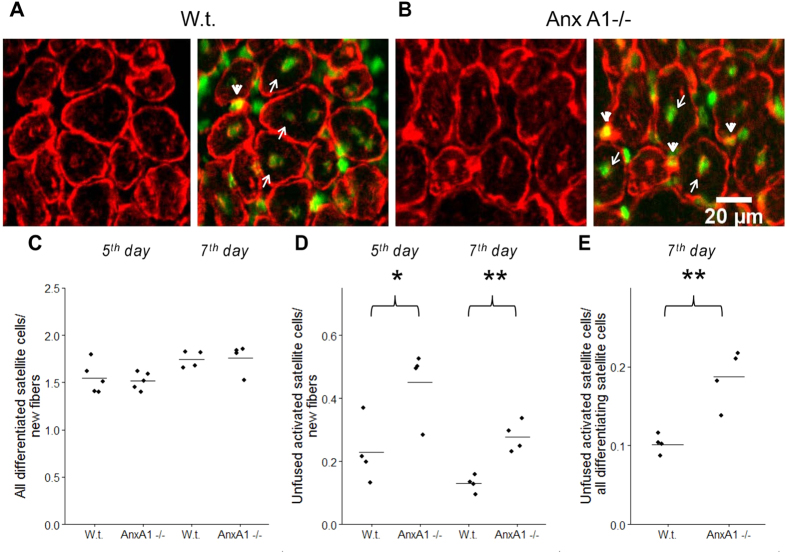
Anx A1 deficiency lowers efficiency of fusion for differentiating muscle cells. (**A**,**B**) Muscle sections taken at 7^th^ day post-injury from w.t. (**A**) and Anx A1−/− (**B**) mice were labeled with desmin (red) and BrdU (green) antibodies to mark differentiating muscle cells and nuclei of newly divided cells, respectively. Arrowheads mark differentiating but yet unfused myoblasts identified as desmin-and BrdU-positive cells located at the surface of myofibers. Arrows mark nuclei of newly generated myoblasts that had already fused and were identified as BrdU-labeled nuclei in desmin-labeled myofibers. Scale bar 20 μm. (**C**) The total numbers of fusion-committed myogenic cells in muscle sections taken at 5^th^ and 7^th^ days post-injury were quantified as the sum of the numbers of fused myoblasts (scored as a number of BrdU-labeled nuclei in myofibers) and unfused myoblasts (quantified as desmin- and BrdU- positive cells located within the myofiber basement membrane) normalized to the number of new fibers. (**D**) The numbers of unfused myoblasts in muscle sections taken 5 or 7 days after injury were normalized to the numbers of new fibers in the same slices. (**E**) The numbers of unfused differentiating myoblasts in muscle sections taken 7 days after injury were normalized to the total numbers of differentiating myoblasts in the same slices. (**C**–**E)** The points show the means for each mouse in a given condition and a line within the group of the points shows the mean of the means for all mice in a given condition. Levels of statistical significance of differences between w.t. and Anx A1-deficient mice are shown as * for p < 0.05; and ** for p ≤ 0.01. The rest of the samples were not significantly different from each other, p > 0.05.

As neither the inflammation nor satellite cell activation appeared to be affected by AnxA1 deficit, we next assessed if fusion of satellite cells to form the nascent myofiber was altered in AnxA1−/− muscle. To identify all activated but unfused myoblasts located at the surface of myofibers we co-labelled muscle sections with antibodies to a myogenic marker desmin (an intermediate filament protein expressed in the differentiating myoblasts and myotubes^28^,^29^,^30^,^31^) and antibodies to BrdU (arrowheads in Fig. 8A,B, also Supplementary Fig. 3). Since we found all nascent myofibers with a basement membrane to be larger than100 μm^2^, we classified all mononucleated desmin positive cells <100 μm^2^ as myoblasts. Additionally, since satellite cells differentiate and fuse into myofibers after undergoing a round of replication, we also labeled myogenic nuclei from satellite cells by BrdU labeling. This labeling strategy would pick all cells with the nuclei of myogenic origin, enabling us to avoid inflammatory cells. However it would also miss any rare myogenic cells that did not divide prior to activation and hence did not incorporate BrdU.

Next, we tested if any BrdU positive macrophages that have invaded a damaged myofiber would appear positive for desmin staining. For this, we tested what fraction of desmin- and BrdU-positive cells also label with the macrophage marker F4/80 (Supplementary Fig. 1). In the unregenerated regions of the 3 w.t. muscles at the peak of inflammatory response (3^rd^ day post-injury), 947 out of 953 desmin-and BrdU-positive cells did not label for F4/80. Similarly out of the 1331 desmin-and BrdU-positive cells in 3 AnxA1−/− muscles, 1314 cells showed no F4/80 staining. These data show that macrophages do not appear as desmin-and BrdU-positive cells, indicating these are myogenic cells.

Desmin is an intermediate filament protein that is a relatively late marker of myogenic differentiation^28^,^29^,^30^,^31^ but it has also been suggested to be detected early in differentiation^32^. Thus to further characterize if the desmin-positive myogenic cells located at the surface of myofibers represent only the late, and not the early stage of differentiation, we compared desmin staining with the myogenic cell marker Myf5, which is expressed very early in myogenic progression and then downregulated^28^. In muscle sections 5 days after injury, 39 out of 179 (21.8%) mononucleated desmin-positive cells from 3 w.t. muscles (64 fields of view in 6 sections) were labeled with Myf5 (Supplementary Fig. 2). From 3 AnxA1−/− muscles (50 fields of view in 6 sections) we found 30 out of 140 (21.4%) mononucleated desmin-positive cells to be Myf5 positive. This demonstrated that 1) the desmin positive mononucleated cells represent both early and late stage myoblasts, and 2) at the 5^th^ day post-injury, lack of AnxA1 does not alter the proportion of myoblasts at early and late stage of myogenesis.

By the time of our analysis of muscle regeneration, some of the newly generated myoblasts have already fused into regenerating myofibers yielding BrdU-labeled nuclei in desmin-labeled myofibers (arrows in Fig. 8A,B). To include these cells into analysis, we deduced the number of myogenic cells that have already fused by counting the BrdU positive myonuclei in the nascent myofibers. This number was then added to the number of differentiated but yet unfused myoblasts (identified as mononucleated desmin-positive, BrdU-positive cells located within the myofiber basement membrane). The sum of these two numbers represented the total number of fusion-capable myogenic cells. The total numbers of fusion-capable cells in muscle sections taken at 5^th^ and 7^th^ days post-injury normalized to the number of new fibers in the same fields of view were indistinguishable between Anx A1−/− and w.t. mice (Fig. 8C) indicating that Anx A1- deficit does not inhibit generation of fusion-capable myoblasts. However, in the muscle sections taken 5 or 7 days after injury, the proportion of unfused myoblasts to that of new myofibers was significantly higher for Anx A1−/− mice as compared to the w.t. mice (Fig. 8D). A lowered efficiency of myoblast fusion in regenerating muscle of Anx A1−/− was also revealed by a higher percentage of unfused myoblasts among all fusion-capable myoblasts (Fig. 8E). Increase in the number of unfused myoblasts without an effect on myoblast generation in the injured muscle of Anx A1−/− mice indicates that Anx A1 deficiency slows down the myofiber regeneration by inhibiting myoblast fusion.

## Discussion

Our results indicate that Anx A1 deficiency does not inhibit muscle growth, cause muscle damage or decrease the ability of injured myofiber to repair. Instead, lack of Anx A1 affects myoblast fusion causing a slowdown in regeneration of injured muscle. Elevated numbers of unfused myoblasts and lowered fusion efficiency of differentiating myoblasts observed in Anx A1−/− mice suggests that regeneration of injured Anx A1-deficient mice is inhibited at the cell-cell fusion stage of myofiber formation, rather than at preceding stages of activation and myogenic differentiation of satellite cells. This identifies an important role of Anx A1 in facilitating myoblast fusion *in vivo*.

The Annexin protein family has originated a billion years ago and evolved independently in all major eukaryotic phyla^33^. Most vertebrate tissues, including muscles, express overlapping arrays of the 13 different annexin members (reviewed in^33^,^34^,^35^). Despite distinct expression profiles and properties^35^,^36^, all annexin proteins share a large conserved “core” domain (~30 kDa) responsible for calcium and phospholipid binding. A potential redundancy of contributions of the different members of annexin family in physiology hinders identification of the specific functions of different annexins^6^,^33^,^34^. Despite varied functions, annexins have been frequently linked with coping with stress and injury. For example, Anx A1 helps plants cope with abiotic membrane stress and signaling^37^,^38^. In animal cells, we and others have documented a role of annexins in facilitating repair of injured cell membrane^12^,^15^,^18^,^39^,^40^,^41^,^42^. In view of this we hypothesized that Anx A1 deficit would compromise the repair ability of injured myofibers. Additionally, we hypothesized that similar to poorly repairing dysferlin-deficient muscle, Anx A1 deficiency will increase degeneration and regeneration of the myofibers resulting in reduced contractile force. We observed reduced contractile force in the fast (EDL) muscle of the Anx A1−/− mice. However, this did not correlate with a concomitant increase in muscle histopathology nor did we observe any effect on the ability of the Anx A1−/− muscle fibers to undergo repair from focal laser injury or lengthening contraction injury. These findings suggest that the presence of Anx A1 is not critical for myofiber repair *in vivo*. However, we cannot rule out the possibility that Anx A1 may contribute to myofiber repair *in vivo*, however in its absence another protein (perhaps a different annexin or annexins^25^) substitutes for its role in repair.

In addition to the *in vitro* role of Anx A1 in cell membrane repair, we and others have documented the involvement of Anx A1 in myoblast fusion *in vitro*^6^,^13^,^14^. Interestingly, while at early stages of myotube formation, fusion between Anx A1-deficient myoblasts proceeds much slower than fusion between w.t. myoblasts, Anx A1-deficient myoblasts do catch up and eventually myotube formation is similar to the w.t. myoblasts^6^. This suggests partial redundancy between annexin functions, a conclusion that is supported by the ability of recombinant Anx A5 to accelerate fusion between Anx A1−/− myoblasts^6^. Based on these findings, we analyzed the role of Anx A1 in myoblast fusion *in vivo* during the initial days of the post-injury muscle regeneration. Published reports on muscle regeneration quantify this process by monitoring the following: a) expression of different myogenic differentiation markers at different times post-injury^43^; b) the size of unregenerated (necrotic) areas in the muscle section^44^, and the number of newly formed (regenerated) myofibers^45^. In addition to employing all of these metrics, to specifically assess the extent of cell-cell fusion without being affected by the proliferation and myogenic commitment of the satellite cells, we also used a combination of different end points to assess myoblast differentiation and fusion. We found that Anx A1 deficiency does not inhibit myogenic commitment but slows down fusion of differentiating myoblasts in regenerating muscle. This *in vivo* finding recapitulates our earlier finding *in vitro* that differentiating primary myoblasts from Anx A1−/− mice express myogenic differentiation markers myogenin and myosin heavy chain similarly to w.t. myoblasts^6^. This suggests that in a living animal Anx A1 is involved in myoblast fusion rather than in pre-fusion stages of the myogenic differentiation.

In the present work we report that the lack of Anx A1 slows muscle regeneration *in vivo*, but it neither blocks myofiber repair nor causes any gross inhibition of muscle development. This finding suggests that during early development another annexin is required for proper myoblast fusion leading to normal developmental myogenesis. Alternatively, in Anx A1 knockout mouse the function of Anx A1 during early development leading to myoblast fusion is taken up by another protein (perhaps another annexin(s)). While our results establish the role of Anx A1 in the process of myogenesis *in vivo*, the specifics of this mechanism and the role of Anx A1 in cell fusion needed for myogenesis remain to be fully understood.

## Methods

### Animals

Methods involving animals were approved by the institutional animal care and use committee of Children’s Research Institute. All the methods were carried out in accordance with the approved guidelines. Animals were maintained in a facility accredited by the American Association for Accreditation of Laboratory Animal Care. Methods involving animals were approved by the institutional animal research Committee and animals were maintained in a facility accredited by the American Association for Accreditation of Laboratory Animal Care. For all experimental procedures adult (4–8 month old) male and female mice were used. Wild type (w.t.) mice (C57BL/6) were obtained from Jackson Laboratory (Bar Harbor, ME). Anx A1 knockout mice (Anx A1−/−)^23^ were purchased from Charles River Laboratories UK Ltd (UK). These animals carry an insertion/deletion mutation that interrupts exon 2 and deletes exons 3 and 4 of Anx A1.

### *In vitro* force contraction

Mice were anesthetized with intraperitoneal injections containing ketamine (100 mg/kg) and xylazine (10 mg/kg). From the right hindlimb, the *Extensor Digitorum Longus* (EDL) muscle was dissected and brought into a bath containing Ringer solution (composition in mM: 137 NaCl, 24 NaHCO3, 11 glucose, 5 KCl, 2 CaCl2, 1 MgSO4, 1 NaH2PO4, and 0.025 turbocurarine chloride) at 25 °C that was bubbled with a mixture of 95%O_2_ and 5%CO_2_. With 6–0 silk suture, the proximal tendon was tied to a fixed bottom plate and the distal tendon was tied to the arm of a force transducer (Aurora Scientific, Ontario, Canada, model 305B). The muscle was surrounded by two platinum electrodes to stimulate the muscle. The optimal length of the muscle was established using single 0.2 ms square stimulation pulses. At optimal length, with tetani 300 ms in duration and at frequencies of 30, 100, 150, 200, 220 and 250 Hz, each separated by 2 min intervals, the maximal force of the EDL was measured and normalized for the muscle cross section area. After measuring the length of the muscle with calipers, the muscle was removed from the bath and weighed. The cross section was calculated based on muscle mass, fiber length and muscle tissue density. Fiber length was determined based on the ratio of fiber length to muscle length of 0.45^46^. The same procedure was repeated for the *soleus* muscle from the right hindlimb, but the stimulation duration was 1000 ms at frequencies of 30, 50, 80 and 100 Hz. The fiber length to muscle length for the soleus muscle was 0.71. Before removing the EDL or soleus muscle from the bath, the muscle was subjected to a protocol of lengthening contractions. At optimal length, the EDL muscle was stimulated at 250 Hz for 300 ms until a plateau of maximal force generation was reached. From this plateau, with the muscle stimulated, the muscle was lengthened over 10% of its length with a velocity of 2 fiber lengths per second after which the muscle was passively returned to the optimal length. This was repeated 9 times with 1 min intervals between the lengthening contractions. The same procedure was repeated for the soleus muscle, but the muscle was stimulated for 1000 ms at 100 Hz and the muscle was lengthened over 20% its length. After removal of the soleus muscle, the mice were euthanized with an overdose of CO_2_ followed by cervical dislocation, and different tissues were collected, weighed and embedded as needed.

### *Ex vivo* cell membrane injury

Biceps muscle were surgically isolated from euthanized w.t. or Anx A1−/− mice in Tyrode’s solution and laser injury was carried out using the microscope and laser injury settings as described^47^ in the Tyrode’s buffer containing 1.33 mg/ml FM1-43 dye. The kinetics of repair was determined by measuring the cellular FM1-43 dye fluorescence. FM1-43-dye intensity (ΔF/F, where F is the original value at time 0) was used to quantify the kinetics of cell membrane repair and represented with intervals of 5 frames.

### Histopathology Scoring

For histological analysis, paraffin-embedded muscles from adult mice described above were sectioned and stained with haematoxylin and eosin (H&E) as far as possible using the TREAT-NMD guidelines for the DMD mouse (http://www.treat-nmd.eu/research/preclinical/dmd-sops/). The images were acquired on the VS120 virtual slide microscope (Olympus USA) using the VS-ASWFL software and UPlanSApo 40x/0.95 objective, and each muscle section was assessed for the following criteria: number of myofibers and myonuclei, myofibers with central nuclei, regenerating myofibers, and inflammatory foci. Minimal Feret’s diameter for myofiber was measured using Image J (NIH) plugin.

### Notexin injection

3 months old w.t. and Anx A1−/− mice were anesthetized with peritoneal saline injections containing ketamine (100 mg/kg) and xylazine (10 mg/kg) as described before . After hair removal of the legs, degeneration/regeneration was induced by intramuscular injection of 30 μg of notexin into the entire left *Tibialis Anterior* (TA) muscle using Hamilton syringe, while the contralateral TA muscle was not injected. Prior to injection, syringe was dipped into a blue tattoo dye in order to label the site of injection. Mice were euthanized at the relevant number of days after injection, and both TA were collected, embedded in OCT (*optimum* cutting temperature) and snap frozen in liquid nitrogen-cooled isopentane. All mice were fed 40 mg/mL of Bromodeoxyuridine (BrdU) in their drinking water starting a day before the notexin injection until euthanasia to label the new formed nuclei.

### Image acquisition

Images were acquired using an inverted fluorescence microscope AxioObserver D1 (Zeiss) equipped with 20 × 0.8 NA Plan-Apochromat objective (Zeiss), LUDL filter wheels on both epifluorescence illumination port and image acquisition side port, CoolLed pE-2 LED illuminator (380, 490, 550 and 635 nm), and Ixon 885 EMCCD camera (Andor). Microscope, filter wheels, illuminator and camera were controlled using Micro-Manager 1.4.13. Fluorescence channels were collected sequentially through a penta-band dichroic mirror (FF 408/504/581/667/762, Semrock) using appropriate single band excitation (FF01-387/11, FF02-485/20, FF01-560/25, FF01-650/13 or FF01-740/13, Semrock) and emission (FF01-440/40, FF01-525/30, FF01-607/36, FF02-684/24 or FF02-809/81, Semrock) filters. Entire area of each fiber section was automatically scanned and imaged using PZ-2000 XYZ Series Automated Stage with Piezo Z-Axis Top Plate (Applied Scientific Instrumentation).

### Immunofluorescence

The slides were fixed in ice-cold acetone 10 min at −20 °C, washed with PBS at the room temperature (RT) and incubated in 2N HCl 1 h at 37 °C to denature the DNA for BrdU staining. After 3 washes with Tris-Buffered Saline (TBS buffer), we neutralized HCl with sodium borate 0.15M for 10 min at RT. Then slides were incubated in the blocking solution (7.5 mL of PBS; 2 mL of goat serum; 200 mg of BSA; 50 μL of Triton X100 and 10 μL of tween 20) for 1 h at RT, incubated overnight at 4 °C with primary antibodies (BrdU + laminin) and rinsed 3 times with TBS. After 1h incubation at RT with secondary antibodies and 3 TBS washes cell nuclei in the slides were stained with propidium iodide (20 min at RT, Sigma, # P4170, stock: 2.5mg/mL in PBS), used in 1/1000 in PBS and washed 3 times with TBS. In some experiments laminin antibodies were replaced with desmin antibodies and PI labeling was skipped and either F4/80 or Myf5 antibodies were used. In the case of Myf5 labeling, we skipped the acid application and replaced BrdU labeling with Sytox Green (Invitrogen) labeling.

All antibodies were applied in PBS with 10% of blocking solution. BrdU was detected with biotinilated primary antibody (Life Technology #B35138) in 1/100 dilution followed with Alexa 488 streptavidin (Invitrogen S32354), in 1/500 dilution. Laminin was detected with primary antibody (Sigma #L9393), in 1/400 dilution followed with the secondary antibody Marina Blue® goat anti-rabbit IgG (Invitrogen #M10992, in 1/500 dilution). Desmin was detected with primary antibody (Abcam, #ab15200, in 1:200 dilution) followed with the secondary antibody Alexa-Fluor 568 goat anti-rabbit Ig (Invitrogen, # GA11011, in 1/200 dilution). Macrophages and Myf5-expressing cells were detected with F4/80 antibody, clone CI:A3-1 (Abcam, ab6640, in 1/50) and anti-Myf5 Antibody, clone 1E2G4 (EMD Millipore Biosciences, #MABE74, in 1/50 dilution) followed with the secondary Goat anti-rat Alexa-Fluo 350 Invitrogen A21093 in 1/300 dilution.

### Analysis and statistics

For muscle histology and membrane repair analysis the data are presented as averaged values for all of the muscles or myofibers used for that analysis (described in figure legends). These averaged values were compared with each other using unpaired Student’s T-test. To assess muscle regeneration, we analyzed muscle sections taken from 4 mice and focused on the fields of view in the vicinity of the injury identified as the fields of view with ≥10 myofibers with centrally located BrdU-labeled nuclei. For each mouse, we examined 35–50 fields of view taken from ≥3 slices. The results for all fields of view in all slices for each mouse were pooled together to find means for each mouse in each condition. These means were then used to analyze the statistical significance of differences between different groups of mice (for instance, to compare 4 w.t. mice and 4 Anx A1−/− mice 5 days post-injury). In analysis of the area of new fibers (identified as fibers with centrally located BrdU-labeled nuclei) we examined >3000 fibers for each condition. Statistical analyses were carried out using the unpaired Student’s t-test or, when the data were not normally distributed or failed the equal variance test, the Mann-Whitney rank sum test in Sigmaplot v.11.0 (Systat Software, San Jose, CA).

## Additional Information

**How to cite this article**: Leikina, E. *et al.* Annexin A1 Deficiency does not Affect Myofiber Repair but Delays Regeneration of Injured Muscles. *Sci. Rep.* **5**, 18246; doi: 10.1038/srep18246 (2015).

## Supplementary Material

Supplementary Information
