# Supplementary material for: Annexin A1 Deficiency does not Affect Myofiber Repair but Delays Regeneration of Injured Muscles
Source: Sci Rep. 2015 Dec 15;5:18246. doi: 10.1038/srep18246 (PMC4678367; doi:10.1038/srep18246)
Supplement: Supplementary Information [file srep18246-s1.pdf]

ANNEXIN A1 DEFICIENCY IN VIVO DOES NOT AFFECT MYOFIBER REPAIR BUT DELAYS REGENERATION OF INJURED MUSCLES

Evgenia Leikina[1*], Aurelia Defour[2*], Kamran Melikov[1], Jack H. Van der Meulen[2], Kanneboyina Nagaraju[2,4], Shivaprasad Bhuvanendran[2], Claudia Gebert[3], Karl Pfeifer[3], Leonid V. Chernomordik[1#], Jyoti K. Jaiswal[2,4#]

[1] Section on Membrane Biology, Program of Physical Biology, Eunice Kennedy Shriver National Institute of Child Health and Human Development, National Institutes of Health, Bldg. 10/Rm. 10D05, 10 Center Dr. Bethesda, Maryland 20892-1855, USA;

[2] Children's National Medical Center, Center for Genetic Medicine Research, 111 Michigan Avenue, NW, Washington DC 20010-2970, USA

[3] Section on Genome Imprinting, Program on Genomics of Differentiation, Eunice Kennedy Shriver National Institute of Child Health and Human Development, National Institutes of Health, USA;

[4] Department of Integrative Systems Biology, George Washington University School of Medicine and Health Sciences, Washington DC, USA

* These authors made *equal contributions* to the work.

[#]**Correspondence to:** Dr. Leonid V. Chernomordik, e-mail: chernoml@mail.nih.gov or Dr. Jyoti K. Jaiswal, email: jkjaiswal@cnmc.org

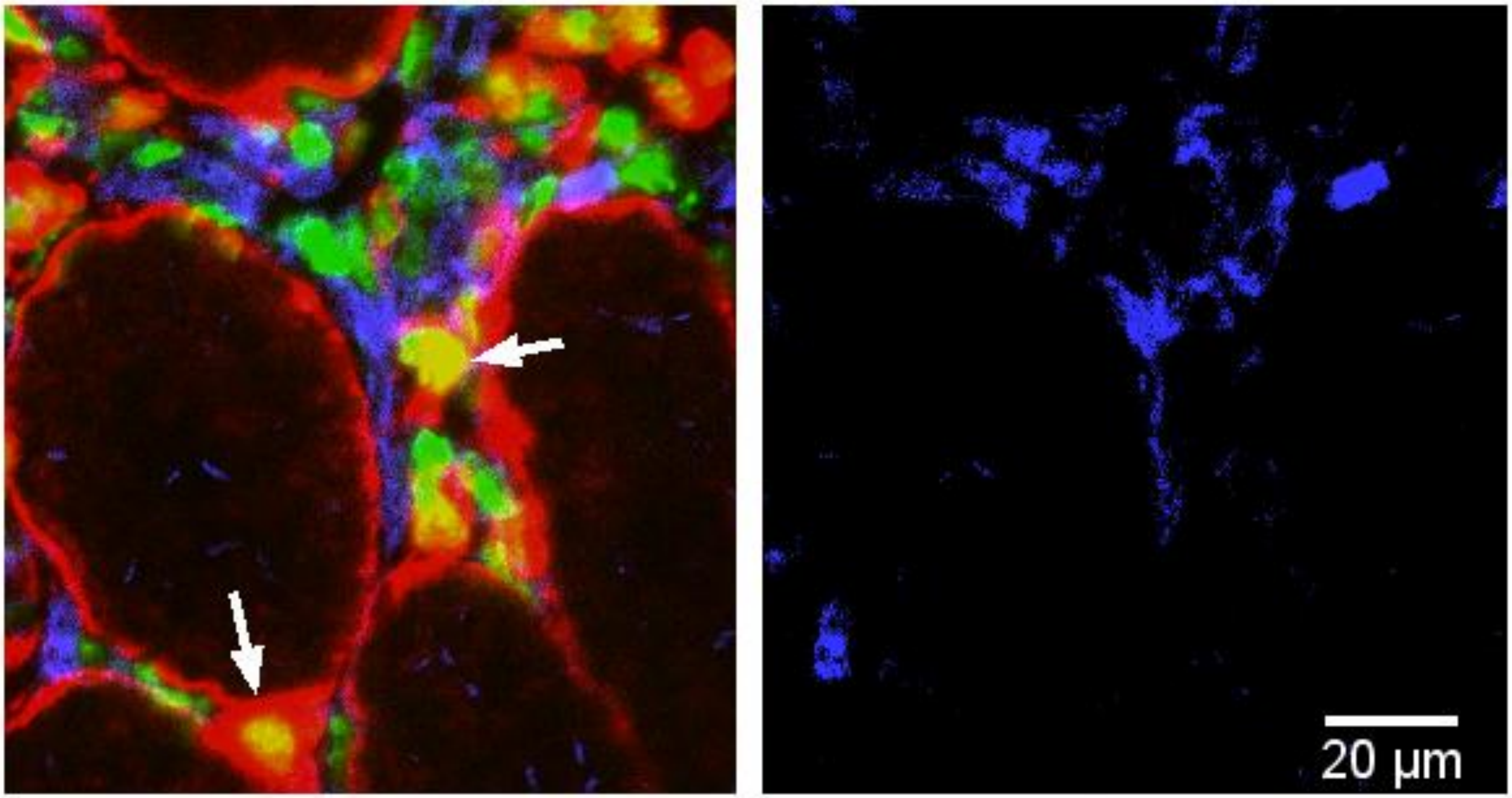

Supplementary Figure 1. Macrophages in the unregenerated regions detected by macrophage F4/80 antibody (blue) are not recognized by desmin antibody (red) and BrdU-antibody (gree) arguing against significant contribution of macrophages to the population of desmin-labeled cells. Arrows show desmin-positive, BrdU-positive cells that are not labeled by F4/80. Unregenerated region of the muscle section of w.t. mouse at the 3rd day post-injury. Scale bar 20μm.

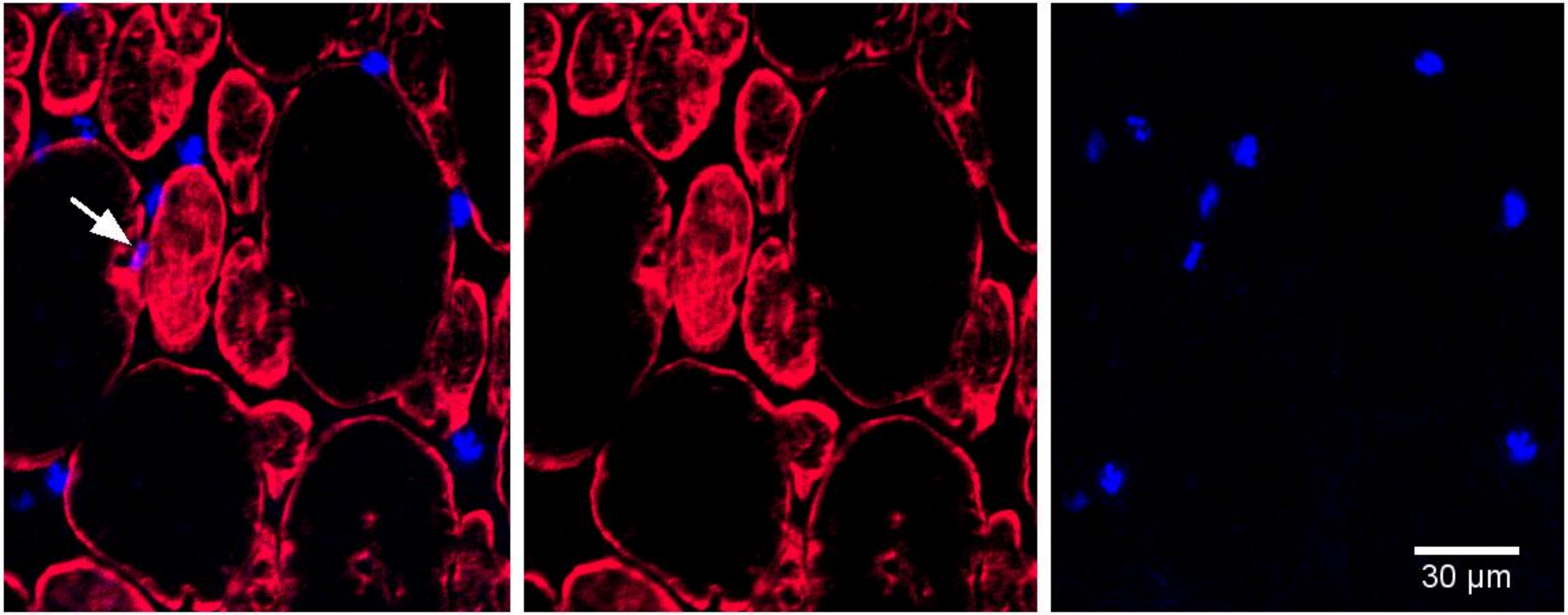

Supplementary Figure 2. Muscle section from w.t. mice taken 5 days after injury labeled with two markers of myogenic regeneration: Myf5 antibodies (blue) and desmin antibodies (red). Arrow marks desmin-positive Myf5-positive cell. Scale bar 30μm.

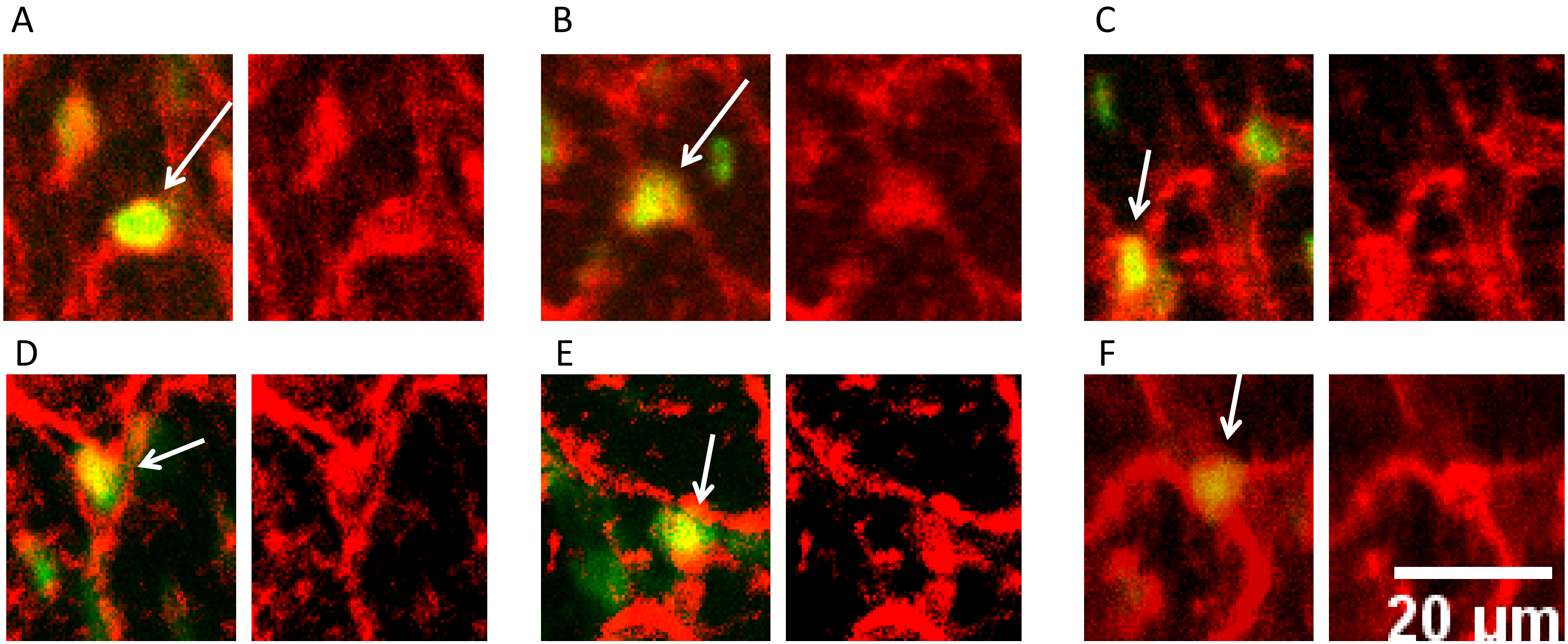

Supplementary Figure 3. Identification of unfused myoblasts (marked by arrows) as mononucleated desmin-positive (red), BrdU-positive (green) cells located at the surface of myofibers. All 6 examples are taken from regenerating regions of the muscle sections from w.t. mice 5 days after injury. Scale bar 20µm.
